# Animal-Origin Food Waste Across Global Supply Chains: Trends, Upcycling Strategies, and Circular Economy Solutions

**DOI:** 10.3390/foods15122202

**Published:** 2026-06-18

**Authors:** Joana Gonçalves, Raquel P. F. Guiné, Paulo Ribeiro, Sofia G. Florença, Luisa Cruz-Lopes, Ofélia Anjos, Da-Wen Sun

**Affiliations:** 1 Centre for the Research and Technology of Agroenvironmental and Biological Sciences, CITAB, Inov4Agro, Universidade de Trás os Montes e Alto Douro, UTAD, Quinta de Prados, 5000 801 Vila Real, Portugal; joanagoncalves@utad.pt; 2 CERNAS-IPV, Research Centre for Natural Resources, Environment and Society, Polytechnic University of Viseu, 3504-510 Viseu, Portugal; p_renato_saraiva_r19@hotmail.com (P.R.); sofiaflorenca@outlook.com (S.G.F.); lvalente@estgv.ipv.pt (L.C.-L.); 3 CERNAS-IPC, Research Centre for Natural Resources, Environment and Society, Polytechnic University of Coimbra, 3045-601 Coimbra, Portugal; 4 CERNAS-IPCB, Research Centre for Natural Resources, Environment and Society, Polytechnic University of Castelo Branco, 6001-909 Castelo Branco, Portugal; ofelia@ipcb.pt; 5 CBP-BI, Biotechnology Research Centre of Beira Interior, 6001-909 Castelo Branco, Portugal; 6 CEF—Forest Research Centre, TERRA Associate Laboratory, School of Agriculture, University of Lisbon, Tapada da Ajuda, 1349-017 Lisboa, Portugal; 7 Food Refrigeration and Computerized Food Technology (FRCFT), University College Dublin, National University of Ireland, Belfield, 4 Dublin, Ireland

**Keywords:** food waste, primary production, food processing, consumption, leftovers

## Abstract

Recently, the problem of food waste management has attracted the attention of producers, processors, retailers, and consumers due to economic, environmental, food safety, and sustainability consequences, affecting the entire food supply chain. This article reviews data on food waste of animal origin at different stages along the production and transformation systems, from an environmental, economic, or social perspective. Results show differences between developed and developing countries. While in developed countries, most waste occurs at the end of the food chain, in developing countries, most waste occurs in primary production and transportation. Food waste is very expressive in production and retail, but also in final consumption in households and food services. Mitigating measures include upcycling, i.e., recovering valuable food components for industrial use with economic and environmental benefits, and alternatives for food waste reutilization. The role of the consumer is unquestionable, particularly when shopping for food for the household or when consuming food in restaurants or canteens. Hence, it is crucial to understand the behaviours leading to food waste as a way to reduce it and implement strategies to effectively reduce food waste at various levels. The role of education, regulation, and policies is pivotal in achieving minimal food waste.

## 1. Introduction

According to the Food and Agriculture Organization of the United Nations (FAO), food loss encompasses a decrease in the quantity or quality of food in the initial stages of the food supply chain, reducing the amount of food available for human consumption. By definition, “Food loss is the decrease in the quantity or quality of food resulting from decisions and actions by food suppliers in the chain, excluding retailers, food service providers and consumers”; it may be related to the overproduction or problems at primary production, storage, and distribution [1]. In contrast, “Food waste refers to the decrease in the quantity or quality of food resulting from decisions and actions by retailers, food service providers and consumers” [1]. Hence, food losses and food waste occur across the whole food supply chain, from production to consumption and discard [2]. Food waste (FW) is a term broadly used to refer to any discard or loss at the various stages of the food supply chain, namely, production, storage, processing, distribution, and leftovers/discards from households and businesses [3,4].

In developing regions, most FW occurs during the early stages of the food supply chain, particularly in primary production, processing, and manufacturing. In developed regions, however, FW is more prevalent at later stages, especially in retail, distribution, and consumption sectors [5].

It has been reported that around 90% of FW in Australia comes from primary production, processing, households, and businesses [3]. FW has not only economic but also environmental consequences, representing a misapplication of resources within the food system, which translates into environmental pollution (emission of gases that contribute to the greenhouse effect) and depletion of natural resources, namely water [3]. Each year, this waste results in a massive carbon footprint, corresponding to the production of 3.3 billion tonnes of CO_2_ equivalent [6]. The world population, currently around 8 billion people, is projected to reach 9.8 billion by 2050, increasing food demand and placing additional pressure on the global food supply system [7].

The increase in FW has been associated with economic growth, urbanisation, and income levels [7]. In addition to depleting natural resources and deteriorating ecosystems, FW will be the main reason for food deficiency in the future, with more than half a billion people going hungry every year [7,8].

Waste of animal products depletes valuable resources such as water, land, and feed beyond caloric loss, further amplifying environmental impacts, namely GHG emissions. In particular, producing meat and dairy products requires intensive resource inputs, making the waste of these products particularly problematic to environmental sustainability. In addition, livestock production contributes to greenhouse gas emissions, and when animal products are wasted, their associated carbon footprint is increased [9]. Addressing waste in the animal product supply chain is therefore critical to food security, environmental protection, and economic sustainability.

Understanding the factors that contribute to FW along the animal product supply chain is a key aspect in finding solutions to minimise the problem. Issues such as poor livestock management, processing inefficiencies, inadequate storage and transport infrastructures, and consumer behaviour all play a role in exacerbating the problem [10].

Animal products, including meat, fish, dairy, and eggs, are particularly vulnerable to waste due to their perishable nature and the complexity of their supply chains. Losses occur at multiple stages, from production and processing to distribution and consumption, leading to significant inefficiencies [11]. According to the estimates of the FAO, 1.3 billion tonnes of food are lost or wasted every year, representing approximately 33% of global food production intended for human consumption. In the case of the meat sector, it is estimated that 20% of the 263 million tonnes produced are lost or wasted, while for fish and seafood, the percentage of loss is higher, 35%, with 8% of fish captured being thrown back into the sea, not being able to survive anymore. In the dairy products sector, 20% is wasted, with the European region alone accounting for 29 million tonnes of dairy products being lost or wasted in a single year. These losses represent a misutilization of resources (water, land, energy, human labour, and capital), with harmful environmental impacts and excessive emission of gases with greenhouse effects, contributing to global warming and climate change [12]. Given the current problem of FW and the consequences it entails, this review examines FW related to animal products across the entire food supply chain, from primary production to household consumption, addressing key challenges, mitigation strategies, and their economic and environmental impacts.

## 2. Bibliometric Analysis of the Sources

**Figure 1:**
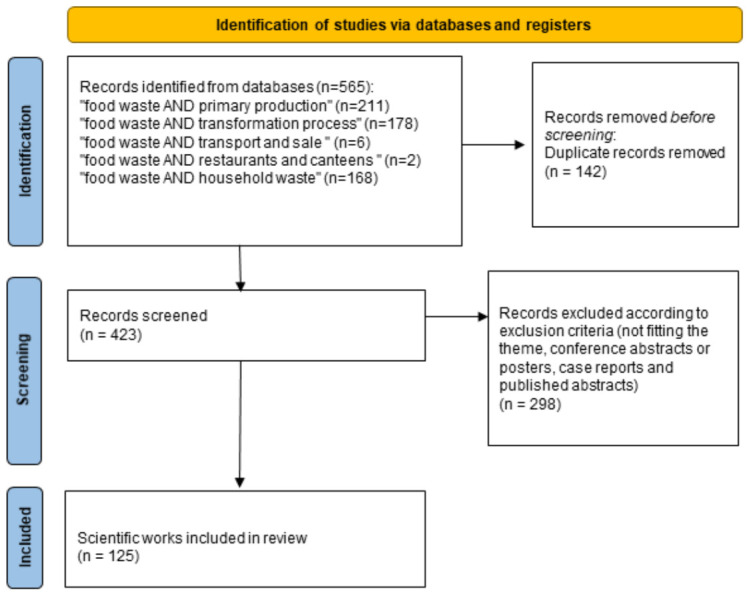
Flow diagram illustrating the different phases of the review.

Figure 1 illustrates the flowchart with the different phases of the review and their respective screenings.

After conducting the bibliographic search, 565 distinct references were obtained. Due to overlap between the different keywords used in the search, 142 of the publications found were duplicates. Thus, 423 publications were submitted to the first screening phase for reading the abstract. After the first phase, 298 studies were excluded according to the exclusion criteria. The remaining 125 publications were submitted to the second screening phase and, after a complete reading of each publication, were all included in this study. Hence, this review is based on a total of 136 sources, of which 125 are scientific articles and 9 are other sources (books, chapters, reports, articles in journals, or conference papers).

**Figure 2:**
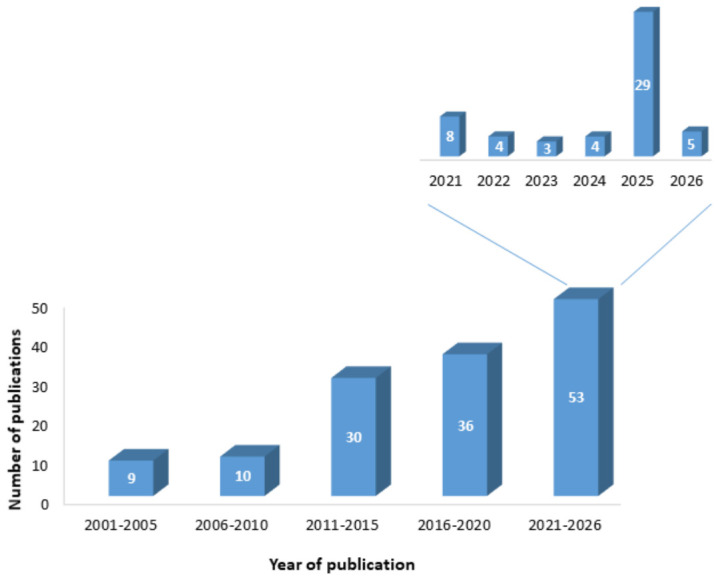
Publication trends of the scientific articles used as sources according to publication year, 2001–2025 (n = 114).

Figure 2 presents the articles by year of publication, showing a great increase in the number of works on this theme over time, expressing the great interest of the scientific community in conducting research related to the topic of FW. The abundance of works in 2025 is expressive, and, although we are still in 2026, there are already some publications in this domain.

**Figure 3:**
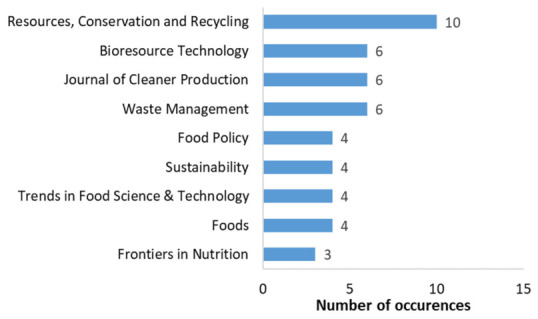
Number of articles according to the scientific journal title.

Figure 3 presents the number of articles according to the scientific journal title, including the most frequent journals, such as *Resources, Conservation and Recycling*. All other journals had only one or two publications.

**Figure 4:**
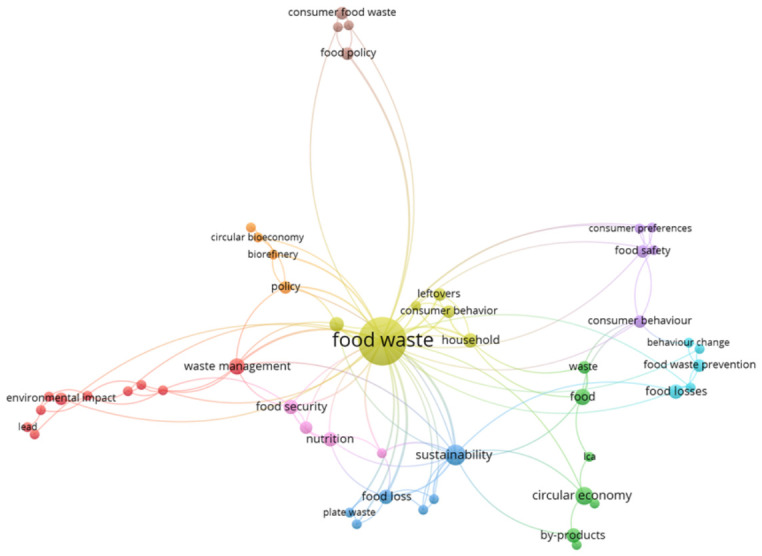
Diagram of co-occurrence links between keywords that occurred at least twice.

The 125 scientific articles were analysed with the software VOSviewer (Version 1.6.20), based on the keywords of each publication. Figure 4 presents a diagram resulting from the analysis of co-occurrence links between the 413 keywords. A co-occurrence threshold of ≥2 was applied, reducing the initial set to 59 keywords; only 51 of them were connected to at least one other keyword, and therefore, those were included in the final diagram. The nodes in Figure 4 have sizes proportional to the relative number of keywords, and the proximity of circles/labels is indicative of sources in which the keywords occur jointly. The most frequent keywords were ‘food waste’ (n = 42 occurrences, with a link strength of 61), ‘sustainability’ (n = 8, with a link strength of 19), ‘food loss’ (n = 4, link strength of 12), and ‘household’ (n = 4, link strength of 10). The 51 keywords were grouped into 9 clusters, with 122 links and a total link strength of 157.

## 3. Food Waste in Primary Production

Distinguishing between the edible and inedible parts of a food is not always easy, and sometimes it can even be subjective. This concept is also related to the different cultures of the world, as what is considered edible in some countries may not be so in others [13]. Thus, it can be difficult to define what is considered FW [13].

Waste generated by livestock farming represents a component of the overall waste stream. However, isolating and quantifying the fraction specifically related to animal products, such as meat, dairy products, and eggs, remains challenging. For some foods, waste generation is unavoidable at certain stages of the supply chain, as in the case of eggshells and animal bones [14,15]. Nevertheless, the amount of waste associated with by-products may be reduced through the implementation of reuse strategies [16]. The reuse of animal-derived food waste as livestock feed has been identified as a promising circular strategy. However, recent evidence suggests that its environmental benefits should be carefully evaluated, as spillover effects may partially offset the expected sustainability gains [17].

Meat production needs have been increasing due to increased consumption related to changes in diet and population growth. Given this increase, it was postulated that by 2020, developed countries would have consumed 107 Mt more meat compared to over 2 decades ago. Likewise, these countries would be responsible for 63% of world meat production [18]. However, in European countries such as the United Kingdom, Sweden, Denmark, Germany, and the Netherlands, a trend towards alternatives to animal protein has developed [19]. In Iran, the per capita meat consumption is around 35.5 kg per year (23 kg of poultry meat and 12.5 kg of red meat), which corresponds to around 100 g/person/day, with the largest meat production being associated with small ruminants such as sheep, followed by cattle production [18]. In contrast, in Turkey, 88% of the red meat produced (3,602,115 tonnes) comes from cattle farming [20]. India is one of the countries that produces and exports the highest amount of beef in the world (10.5 Mt annually), just after China, the United States, and Brazil [21], although this production is almost entirely water buffalo meat (known as carabeef) and not cow beef.

It is estimated that 1.4 billion hectares of agricultural land will produce goods that will end up in waste, with 78% of this wasteful occupation being due to meat and milk waste. Approximately 20% of the meat produced for human consumption ends up being wasted, which has a cost of 750 billion to 1.0 trillion USD in economic losses every year. In Iran alone, an estimated 300,000 tonnes of meat are wasted annually, which is still 15% less than the amount wasted globally [18]. Despite this, meat waste represents only 7% of total FW, less than grains or fruits and vegetable waste, even though this percentage results in a 20% GHG impact [18]. Recent analyses of livestock systems further confirm that beef and dairy production are among the largest contributors to greenhouse gas emissions in animal-based food systems [22]. In fact, the livestock sector is responsible for 70% of land use for pasture, 80% of agricultural land occupation, and 18% of greenhouse gas emissions. Therefore, this sector causes more environmental problems compared to chicken farming, which emits fewer greenhouse gases [23].

Most meat waste generated is produced during slaughter in slaughterhouses, although it can also occur in the early stages of production in animals that are unsuitable for slaughter or that have died [21,24]. During slaughter, waste is generated, such as tendons, skin, bones, blood, internal organs, and gastrointestinal contents, which cannot be sold either as meat or as meat products [21]. Waste in slaughterhouses can also be generated by excessive or unnecessary trimmings, poorly adjusted equipment, or inadequate technical practices [18]. Meat by-products represent around 68% of live weight in lambs, 52% in pigs, and 66% in cattle; although part of these by-products is reused, increasing the profitability of meat (more than 11.4% in cattle and more than 7.4% in pigs), another part (approximately 50%) is not suitable for human consumption given its characteristics [21]. In a bovine slaughterhouse, 275 kg of waste is produced per ton of live weight (27.5% of the animal’s total weight). In just one year, 35 Mt of meat and 14 Mt of by-products were produced in the European Union [19]. Also, in Turkey, in 2020, approximately 18 kt of blood and 41 kt of bones were generated as by-products from meat production [20]. In the United States of America, these by-products are divided into edible and inedible. Edible products are then processed under controlled sanitary conditions and include blood, meat trimmed from the head, and edible fats obtained during slaughter, which include fat from the bonnet, back, or rumen [21]. On the other hand, in the United Kingdom, the head, liver, tail, tongue, lungs, fat, viscera and bladder, rumen, feet, and trimmings are considered edible by-products. In the case of poultry, the necks, heart, liver, and gizzards are also edible by-products [21]. Blood is a by-product of meat production that has also been consumed over the years. In Europe, it is used to make bread, biscuits, blood sausages, and blood pudding, while in Asia, it is used to make blood cake and blood curd, in addition to blood pudding [25]. Blood is rich in proteins and can be considered sterile when obtained from a healthy animal. Therefore, this by-product is also used in feed, blood meal (for cattle), fertilisers, and binders [21]. Blood plasma has an excellent ability to bind water and fat and form a gel. Additionally, it can form foam, which is used in the baking industry as a substitute for egg whites [21]. Gelatin can be extracted from the skin and leather of animals and can be used for various food purposes, including transformation into jellies, gelatinous desserts, frozen desserts, ice cream stabiliser, yoghurt, or meat pies. Pig skin can also be used in the production of cracklings after being soaked, boiled, dried, and fried. Bones can also have other uses. Different animals have different percentages of bones; 16% of the weight of a lamb, 11% of the weight of a pig, and 15% of the weight of a cow correspond to bones, and sometimes, there is still some meat attached to these [21]. For many years, bones were used to extract gelatin; however, currently, attempts have been made to obtain the largest quantity of mechanically deboned poultry meat (MDPM), which is approved for consumption in meat products such as hamburgers, ground meat, and sausages, although with some restrictions [21]. In Australia, MDPM in exported products must display on the label the quantity present, moisture, protein, and calcium content. In the United States of America, this product cannot be used in baby food, minced meat, burgers, or meat pies. In Denmark, if MDPM is present in an amount greater than 2%, it must be stated on the label [21].

**Figure 5:**
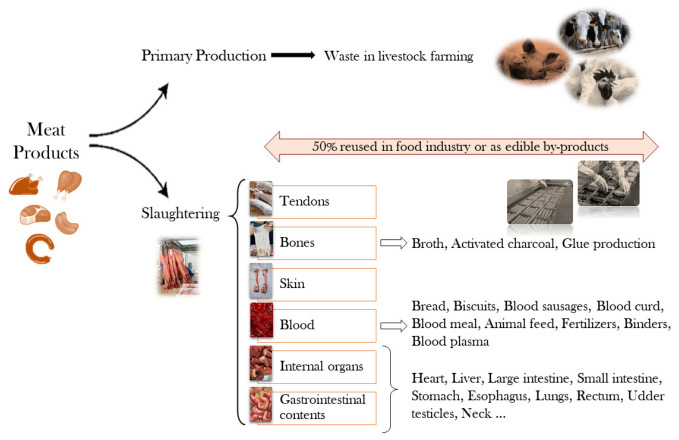
Waste from the meat sector.

Figure 5 summarises the different meat wastes generated in the sector.

In addition to meat, there are other products of animal origin that also end up being wasted. Eggs are a product that is widely consumed worldwide and has several applications in food production and cooking [13]. About 540,542 t/year of eggs are consumed in Korea [13]. Given their widespread use, large amounts of waste are also produced worldwide [13]. In North America, around 31.4% of eggs produced were wasted along the supply chain [26]. Also, in Switzerland, a significant number of eggs are wasted [27]. The largest percentage of this waste occurs on the part of consumers (64%), but 9% occurs during retail, and 18% during production [27]. In fact, eggs are physically vulnerable products and are also vulnerable to attack by microorganisms, which are present in the faecal traces that sometimes remain on the shell. If the shell is not quickly and properly cleaned, these microorganisms may spread [28]. Eggshell residues do not yet have any implemented alternative application; however, studies suggest that they could be used as immobilising agents for heavy metals, namely Cr(III)/Pb [29,30].

Another animal product that generates some waste is milk. This product is of great global importance, but its production also has some impact on the environment [13]. In Mexico, between 3.74 and 11.22 million m^3^ of milk waste is generated annually. Even so, it is expected that the agricultural stage is where the greatest production of milk residues occurs [31,32].

**Figure 6:**
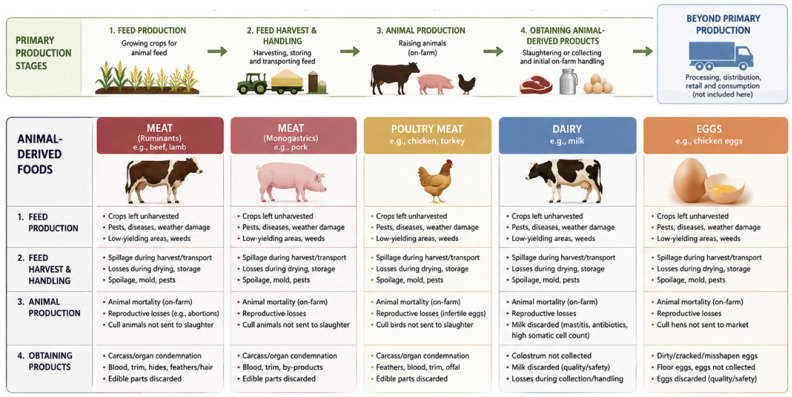
Food waste in the primary production of animal-derived foods.

Figure 6 shows the food waste in the primary production of animal-derived foods, including meat products, dairy, and eggs, and focuses on wastes occurring on farms before slaughter or collection, i.e., from raising animals and producing feed to obtaining animal-derived foods.

Fish is a highly sought-after food and one of the most traded in international markets [13]. Fish is a source of protein, contributing around 17% of the consumption of protein of animal origin worldwide, and 6.5% of all its consumption, i.e., including all sources of protein. Additionally, fish is a source of other macromolecules such as cholesterol, carbohydrates, and micronutrients (vitamins, minerals, and polyunsaturated omega-3) and has a low content of saturated fats [13]. Therefore, since several health benefits are associated with the consumption of fish and seafood, their consumption has been increasing worldwide [13]. In Australia, an average of 25 kg of seafood was consumed per capita in just one year. This data indicates that demand and consumption of these foods have been increasing, given that in the 1970s, an average of 13.6 kg was consumed [33]. However, this increased demand raises sustainability concerns, as inherent to this increase is increased waste [13]. Waste associated with fish and seafood and their mismanagement can reach a cost of 50 billion USD each year [34,35]. Various types and quantities of fish waste are produced throughout the supply chain, with fishing and aquaculture alone accounting for 130 Mt of waste per year globally [36]. Waste produced during fishing mainly results from the accidental or unintentional capture of a certain species and is subsequently discarded. However, there is no effective solution to this problem, with an estimated 17.9–39.5 Mt/year of whole fish being discarded [36]. However, studies indicate that this waste can be greatly reduced if appropriate and sustainable changes are implemented [37]. It should be noted that there are still many fishing activities that are not monitored, so the amount of waste generated may be underestimated [37]. Therefore, strategies to minimise harm associated with FW are important. In fact, recent studies have highlighted the importance of circular bioeconomy approaches and improving resource efficiency across all agri-food systems to minimise waste generation and increase the recovery of by-products throughout the supply chain [38].

**Figure 7:**
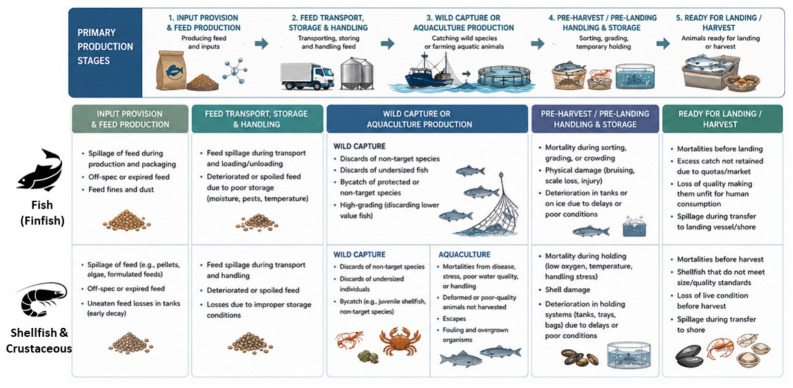
Food waste in the primary production of marine foods of animal origin.

Figure 7 shows the food waste in the primary production of marine foods of animal origin, including fish and shellfish, and focuses on wastes occurring in the wild capture fisheries and aquaculture before landing/harvest, from input provisioning to animals ready for human consumption.

## 4. Food Waste in Food Processing

Waste associated with the transformation and processing of animal products, including meat, dairy, eggs, fish and seafood, is produced by increased industrialisation [39]. In the meat industry, parts of the animal that are not commonly consumed, such as offal, hooves, and certain fats, are often wasted unless they are reused for animal feed, fertiliser, or biofuels. Research shows that slaughterhouse waste consists of the parts of an animal that cannot be sold as meat or used in meat products, including bones, tendons, skin, gastrointestinal contents, blood, and internal organs [40].

Industrial processing generates primary (organic) and secondary (packaging) wastes. Primary waste is composed of proteins, carbohydrates, and lipids, among others, originating from the various processing sectors [41]. Primary waste is obtained in the form of bones, blood, intestines, feathers, or other animal organs [42,43]. On the other hand, secondary waste includes wastewater or packaging material [44]. Another problem is related to the leaching of nutrients that harm aquatic ecosystems, altering the taste and odour of drinking water or promoting the deoxygenation of water that culminates in the death of aquatic beings [41].

The amount of waste produced worldwide by different sectors of the food industry is high [45]. In the European Union, waste generated in food manufacturing amounts to around 367 Mt/year, and the food processing market is expected to reach 4.1 trillion USD by 2024 [46,47]. Countries such as Germany, the United Kingdom, Italy, France, and Spain contribute the most to the generation of these quantities [48]. Also in India, around 600 tonnes of agro-industrial waste are produced annually [41].

Food processing allows for extending shelf life and offers consumers alternative food products. There is also a wide variety of foods that cannot be consumed raw, requiring some processing before they are suitable for human consumption. Thus, during processing, a preservation process is induced in food, increasing its shelf life [46].

One of the transformation sectors that generates high levels of waste is the fish processing sector. Some recoverable compounds from fish waste streams include protein from fish muscles, skin, fins, bones, heads, viscera, and scales (49–58%) and collagen from fish skin and scales (5–51%) [49]. Also, the dairy industry produces streams that can be used to recover some valuable compounds, like protein (0.6–0.8%) and lactose (4–5%), both from milk whey [50].

Table 1 shows some works focusing on FW during the processing of animal-based food products. Strategies such as upcycling and recovering components and substances from FW for high-value industrial applications in a circular economy context are highlighted as the most suitable to mitigate the impact of FW, while also generating economic value.

Recent studies further highlight the diverse and promising approaches to the sustainable valorisation of food waste, including biological, thermal, chemical, and mechanical processes designed to recover high-value compounds and reintegrate them into industrial production systems [58]. A recent systematic review further emphasises that the conversion of food loss and waste into livestock feed represents one of the most promising valorisation pathways, particularly due to its potential environmental benefits and contribution to reducing feed–food competition [59]. Microbial and enzymatic recovery approaches have also received considerable attention in recent years as sustainable alternatives to conventional disposal methods. Several microorganisms and enzymes have been used at an industrial level for the conversion of animal-derived food waste into biofuels, biosurfactants, bioplastics, and biofertilizers, simultaneously contributing to waste reduction and greenhouse gas mitigation [60].

### 4.1. Processing of Foods of Animal Origin

The meat processing industry generates a variety of wastes in considerable quantities [46]. In the European Union, during 2016, 14 Mt of by-products were produced during the processing of 70 Mt of beef [41]. Australia produces waste during meat processing that represents about 4% of the animal’s mass. In the United States of America, animal processing results in the production of waste worth 69 billion USD in poultry and 83 billion USD in meat [41]. In Denmark, meat processing produces 34,000 tonnes of waste, some of which is inedible [24]. Indeed, Europe has large amounts of wasted meat. In the United Kingdom, 56% of meat and fish is wasted during processing; in Portugal, Spain, France, the Netherlands, Belgium, Finland, Denmark, Sweden, Germany, Poland, Hungary, Italy, and Greece, that percentage varies between 35% and 42% [13].

Waste during meat processing raises many economic, profitability, food safety, and sustainability concerns [13]. Industrial meat waste occurs mainly due to mismanagement, inappropriate use or faulty machinery, poor production methods, and inexperienced or unskilled workers. Additionally, meat is also exposed to biological risk, namely contamination by pathogenic microorganisms such as *Escherichia coli*, *Salmonella enterica*, *Listeria* spp., *Campylobacter* spp., and *Yersinia enterocolitica*, as well as prions [61]. Despite constant technological advances, many slaughterhouses remain non-mechanised or only partially mechanised, which also contributes to meat losses [18].

The main poultry waste is generated by mechanically deboning meat, consisting of losses from the neck, back, and meat attached to the bones, which represents 88.33% of total poultry losses [62]. On the other hand, the most generated by-products in red meat processing are skin and contents of the gastrointestinal tract, blood, bones, tendons, and internal organs [13]. Other waste products such as feathers, hooves, and horns have also been described [46]. However, the use of by-products from meat processing is not always possible, since there are regulatory requirements, which vary between countries, that restrict this use for reasons of quality and food safety or require the declaration on the label of the by-products used. This is the case with sausages and mortadella, when they include mechanically separated poultry meat or heart meat. Although not always approved for consumption, meat by-products such as lung, kidney, brain, spleen, blood, liver, and tripe have good nutritional value [21]. By-products such as cattle tail, ears, feet, and liver have a significant amount of collagen and a protein content similar to that present in lean meat [63]. On the other hand, brain and adipose tissue have lower protein values [21]. Regarding the quantities of amino acids, ears, feet, lungs, stomach, and tripe have lower quantities of tyrosine and tryptophan and higher quantities of proline, hydroxyproline, and glycine. Regarding vitamins, some waste has appreciable values, namely riboflavin (kidney and liver), niacin (liver), vitamin B12 (kidney and liver), B6 (kidney and liver), folacin (liver), ascorbic acid (liver), and vitamin A (liver) [21]. Some offal also has good levels of iron, copper, manganese, phosphorus, potassium, and sodium. Polyunsaturated fatty acids, cholesterol, and phospholipids are also present in the viscera, and monounsaturated fatty acids are present in small quantities [21]. The presence of cholesterol and the possibility of pesticides, drug residues, and toxic heavy metals are reasons why consumption is recommended to be limited [21]. In addition to viscera and offal, animal fats are also by-products obtained during meat processing, the most common being lard and tallow. These two products come from the adipose tissue of animals and can be used as edible fat. Traditionally, they were used for frying, although there are now alternative options. They can also be used in the production of emulsified products and sausages. Blood has good potential in the pharmaceutical industry, which is why it has also been reused and used for this purpose [21].

Livestock blood waste, which is rich in proteins, can be used to recover biogas as well as other valuable by-products through anaerobic digestion. Advanced techniques allow for the mitigation of the environmental impact of blood waste while promoting the circular bioeconomy. Additionally, the possibility of using blood waste from slaughterhouses for the recovery of valuable products such as proteins, heam iron, and bioactive peptides has both economic and environmental advantages [55].

Meat processing is not the only process that generates waste. Large amounts of wastewater are generated, containing residues of fat, protein, or blood, which are therefore rich in organic matter [46]. Poultry production also involves the use of antibiotics and additives, which are then excreted by the animals, contaminating the soil. Thus, some adverse effects on human and animal health and the environment are related to poultry production, since chicken litter contains contaminants such as viruses, growth hormones, antibiotics, heavy metals, parasites, and bacteria [64].

Industrial egg production, particularly in the production of frozen, liquid, and powdered eggs, generates a considerable amount of eggshells [47]. This waste must be managed efficiently, since the growth of this sector of the industry depends on good waste management [13,21]. Egg processing by-products contain a quantity of flavoprotein, which can be used as a good food ingredient [21].

Another product of animal origin that is highly processed at an industrial level is milk. This product is transformed into other products that are in high demand, namely cheese, butter, powdered milk, drinking milk, condensed milk, yoghurt, and ice cream [65]. Australia is a major milk processor, exporting a number of dairy products [3]. Also, in Mexico, milk processing generates between 3.74 and 11.22 million m^3^ of waste annually, and in Denmark, 71,000 tonnes of dairy waste are produced each year [13,41]. Also, in Portugal, Spain, France, the Netherlands, Belgium, the United Kingdom, Denmark, Germany, Sweden, Finland, Poland, Italy, Hungary, and Greece, between 43% and 48% of waste was generated during the industrial processing of milk [13]. This processing results in a large quantity of lactose-rich whey (in Australia alone, around 1.6 Mt), resulting from the coagulation of milk proteins [3,47].

The dairy industry generates large amounts of wastewater (2.5 L to 3 L for each litre of milk processed), which is rich in organic compounds, namely protein, fat, sugars, and traces of detergents [46]. This waste may also contain additives and preservatives with nitrogen concentrations of up to 830 mg/L [46].

Fish processing generates a large amount of waste, which mainly consists of the head, fins, scales, bones, and internal organs of the fish [3]. After capture, this is the stage where most fish waste occurs [13]. In the fish industry, only fillets are used, with the remaining 66% being thrown away [66]. Seafood also generates a considerable amount of waste in the form of shells, shrimp shells, and crustacean endoskeleton [46]. Thus, the seafood industry produces between 6 and 8 Mt of waste every year [46]. This amount of waste corresponds to between 50% and 70% of the initial product, with the seafood processing industry being responsible for 20% of the world’s food supply [46]. In Australia, this sector of the industry produced 20,000 tonnes of fish waste annually, with each ton costing 150 USD to dispose of [13]. Also, in East Africa, large volumes of waste are produced annually from the processing of Nile Perch [13]. In the United Kingdom, on the other hand, around 50% of cod is wasted during processing. Although each ton of this fish costs the processor 3.129 USD, only 63 USD is generated as a by-product, and its disposal can cost 94 USD [13]. In the case of shellfish, only 43% is consumed, with the remainder being discarded [34]. In Portugal, Spain, France, the Netherlands, Belgium, the United Kingdom, Finland, Sweden, Denmark, Poland, Germany, Hungary, Greece, and Italy, the waste of shellfish varies between 40% and 70% [13].

In fact, processing is the largest contributor to waste generation [13]. In East Africa, 36,000 tonnes of solid waste and 1,838,000 m^3^ of wastewater are produced annually [13]. These two waste products are rich in valuable nutrients [67]. Oil from fish waste is also a waste rich in omega-3 fatty acids, and fish skin and scales are rich in collagen [3]. Waste from seafood processing also has a significant content of organic constituents, namely chitin [68]. The disposal of this waste, in addition to having a high monetary cost, also has a major environmental impact [13]. Therefore, more sustainable alternatives must be adopted. Fish waste can be transesterified to generate biodiesel [3]. Likewise, the collagen present in the waste can be used as a food additive or biomaterial [49].

### 4.2. Problems Generated by the Incorrect Disposal of Industrial Food Waste

The industrial processing of food products generates a significant amount of waste [46]. This waste comprises high quantities of biodegradable organic substances such as proteins, fat, or carbohydrates [69].

One of the most significant environmental impacts of improper disposal is the emission of greenhouse gases, particularly methane (CH_4_), which is released when organic waste decomposes in landfills. Methane is a greenhouse gas with a global warming potential more than 25 times that of carbon dioxide (CO_2_) [40]. Beyond emissions resulting from waste decomposition, food waste also represents a substantial loss of embedded energy throughout the food supply chain. For example, the U.S. food system was estimated to consume 7130 TBTU of primary energy and generate 970 million metric tonnes of CO_2_-equivalent emissions annually, with on-farm production, food consumption, and food manufacturing accounting for the largest shares. These figures illustrate the broader environmental burden associated with inefficiencies in food production and waste generation [70]. In addition, leachate from decomposing animal waste can infiltrate soil and water sources, contaminating groundwater and surface water with harmful pathogens and nutrients such as nitrogen and phosphorus. This can cause eutrophication, which depletes oxygen levels in aquatic ecosystems, leading to fish kills and reduced biodiversity [71].

Waste from the food industry can cause health complications, including diarrhoea, drowsiness, tremors, rapid heartbeat, nausea, kidney, respiratory and immunological complications, decreased sperm quality, and changes in the nervous system that can lead to permanent brain damage [46]. This is because contaminants such as mercury, trichloroethylene, arsenic, and benzene are present in this waste [72]. Pollutants present in industrial waste affect marine life, interfering with the reproductive physiology of fish and the sex ratio in embryos, and may even cause death [73,74].

**Figure 8:**
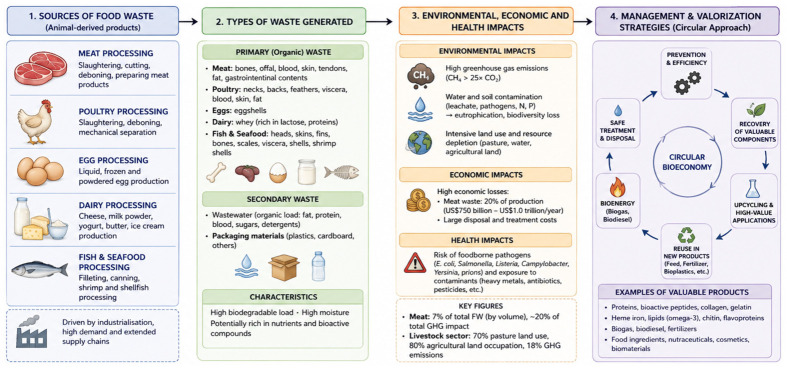
Food waste of animal origin at food processing stage.

Considering the diverse negative impacts of the misuse of industrial FW, it is necessary to apply and manage this waste, redirecting it for recycling and the production of value-added products [75]. Some residues, namely those from fish, can be used in the production of dietary products, natural pigments, or cosmetics [21]. Figure 8 summarises FW from different food sources, as well as its environmental, economic, and health impacts.

## 5. Food Waste in Transportation and Sale

FW in transportation and sale occurs due to improper handling, inadequate storage conditions, and inefficiencies in the supply chain, leading to significant amounts of perishable food being discarded before reaching consumers [76]. A large amount of meat loss occurs in developed countries due to standardisation of expiration dates and defects in supply organisation and packaging [18].

It is estimated that worldwide, 263 Mt of meat is lost or wasted, which is equivalent to 75 million cows. Raising awareness of the causes of these losses will help reduce them significantly. In some countries, such as Iran, fresh meat is sold in local markets without packaging, labelling, or processing [18]. In these situations, meat exposed to air tends to dehydrate, discolour, and spoil. Therefore, meat losses occur due to microbial, chemical, and physical causes. In Iran, the cold storage capacity for meat is not uniform; while some provinces have this facility, other, poorer provinces do not have access to such facilities. This is one of the main causes of loss in developing countries [18].

**Figure 9:**
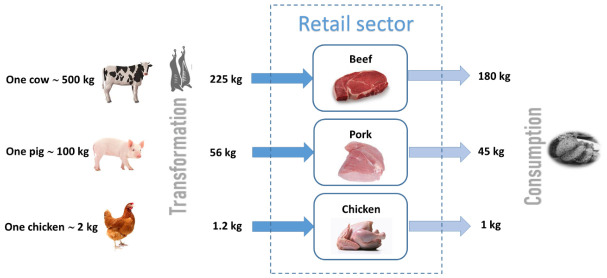
Estimates of losses in the retail sector for some types of meat. Adapted from Pinto et al. [77].

Uhlig et al. [5] report that the retail and distribution sectors are major contributors to food loss and FW along the supply chains. Foods like meat, for example, have a great environmental impact, even when at relatively low wastage volumes, due to the release of gases with a greenhouse effect and intensive use of resources for their production. Figure 9 shows the losses in the retail sector for various types of meat, highlighting that this sector is responsible for 9%, 11%, and 10% losses in relation to live weight for beef, pork, and chicken meats, respectively. However, when looking at local values of input and output weights, the percentages nearly double to 20% for both beef and pork, and 17% for chicken meat.

The expiration date system, although intended to ensure consumer food safety, can also contribute to FW. Consumers often interpret the approaching date on the label as indicating unqualified or unsafe food, leading to waste. The arrangement of products by date on supermarket shelves also leads to waste, as consumers prefer fresher products to the detriment of less recent ones, which are still good for consumption [18]. Waste can also arise from buying in excess due to discounts, for a specific recipe or occasion, without specifying quantities [78]. Consumers may also sometimes fail to understand the concepts of expiration and other information on labels, ending up wasting meat that is still of eating quality, which is why these concepts should be standardised [79]. Since meat has a shelf life of between 7 and 26 days, once the expiration date has passed, unsold products are immediately thrown away, which is the main reason for waste in retail [80]. Another reason is related to the size of the packaging [13]. Recently, new, more technological strategies have been implemented to combat food waste. The creation of smart labels and sensors that indicate product freshness based on colour changes is one of them. There are also smartphone apps that allow for label reading and subsequent analysis of food freshness, making the process more efficient [81].

In order to commercialise beef products in a timely and profitable manner, it is important to successfully manage the beef colour in retail. While a vivid cherry red colour is associated with freshness and quality, discolouration can lead to consumer rejection [82].

Eggs are a food item that suffers from waste if the environment during transport, distribution, and storage is not suitable [13]. In the United States of America and Switzerland, 9% of all egg waste is produced in the retail sector [14,27].

Dairy products are another animal product that can generate some waste at this stage of the supply chain due to a lack of appropriate environment and support, and a lack of refrigeration during transportation. Also, slow sales can contribute to the waste of these perishable and fragile products [13]. In the United States, retailers waste 9% of all dairy products [14]. Studies carried out in Spain and the United Kingdom describe that expiration dates, poor product quality during the summer, poor forecasts, slow sales, refrigeration problems during transport, packaging errors, and breakages that occur at the retail stage are the main causes of milk and dairy product waste [80,83].

Over the years, retailers have made efforts to reduce FW and to demonstrate this interest to the public. In the first six months of 2013, one United Kingdom retailer reported that 28,500 tonnes of food was wasted, corresponding to 0.87% of sales volume [83]. The most common reasons for food loss during retail are apparent defects, expiration of the shelf life, breakage or damage to packaging (caused by customers or employees), inadequately prepared items, or excessive stock [16,83]. Failure to meet strict visual and quality standards, namely shape, weight, and appearance, all contribute to the occurrence of waste during retail [16]. Food loss also appears to be influenced by urban or rural environments and geographic regions.

Donating food to social services is a measure to prevent FW; however, only a small portion is donated for this purpose [83]. Data collected from a group of supermarkets showed that 7% of the food that would otherwise have been wasted was donated. Of the donated food, 16.4% was dairy products. On the other hand, 25% of these outlets donated 10% of the lost food to social services, with 17% donating 40% of dairy products [83].

Price reductions for products that would otherwise be discarded, such as approaching expiry dates or minor visual defects, also provide an alternative to FW generated during retail. Dairy products discarded after price reduction had an expired expiration date, unlike those with a normal price, which were discarded due to damage to the packaging [83].

## 6. Food Waste in Food Service Providers

In developed countries, the number of meals eaten in restaurants, whether for consumption on the spot or to take away, has been increasing [16]. The population has been increasingly turning to factories, supermarkets, and restaurants for meals. Thus, the quantities and types of FW generated have also changed [84]. In the catering sector, the main animal FW consists of eggs, seafood, or dairy products [3]. However, the hospitality in some restaurants generates substantial organic waste, including leftover meals and plate waste, which often ends up in landfills [18,85]. The type and amount of waste generated, through leftovers for example, is variable according to food type, although typically, foods such as meat are rarely wasted [86].

In restaurants and hotels, FW can arise from the over-purchase of food items based on inaccurate forecasts [80,87]. In addition, health and safety regulations prevent restaurants and canteens from reusing or recycling food once it has been served. Expired ingredients, unused prepared food, and leftovers often have to be discarded to meet hygiene standards [88].

The main measure that can be applied to surplus food is donation [89]. However, in the case of restaurants, food donation is limited, since only food suitable for human consumption can be donated, so there are biosafety and food hygiene requirements that must be met [88]. This way, leftovers can be taken away by the customer so that they can be consumed later. Additionally, unused food can be donated to charities to be distributed to homeless people and other people in need [18]. In the United States, since the 1960s, there have been food rescue programmes and food banks that collect surplus food from restaurants and supermarkets and distribute it [89]. This is a measure to combat FW that is currently implemented throughout the world, but could still be expanded widely [89]. Food donation combats food poverty by enabling poorer people to access foods such as meat, which are sometimes considered a luxury [18]. Despite this positive action and the existence of these charitable organisations that raise public awareness about the increase in poverty and FW, it is not a solution for all food needs [89]. Although food donation remains one of the most widely adopted strategies for managing surplus food in the food service sector, recent studies emphasise that source prevention remains largely neglected by food system actors and is still insufficiently integrated into public policies addressing food waste. While food redistribution can generate important social and environmental benefits, some authors argue that it should not be viewed as a standalone solution, but rather as a temporary measure that does not address the structural causes of food surplus generation [90]. Despite the solution presented and the social aspects involved, FW must be reduced as much as possible, among other reasons, because of the environmental impact of greenhouse gas emissions [91].

**Figure 10:**
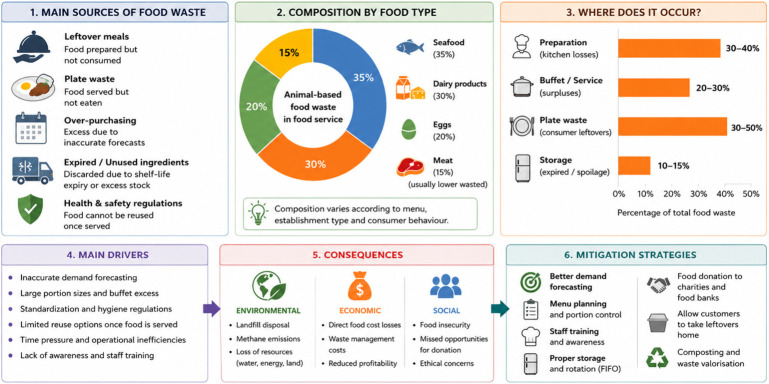
Schematic overview of food waste generation, impacts, and mitigation strategies in food service providers.

To provide a clearer overview of food waste in food service providers, Figure 10 presents a conceptual framework summarising its main sources, key drivers, associated impacts, and potential mitigation strategies. This includes food waste in restaurants, hotels, canteens, and catering services.

Abreu et al. [92] analysed FW in a Portuguese canteen and observed high FW values (due to surpluses and leftovers), varying from 22.1% to 43.6% depending on the school level, preschool or basic school, respectively. In the same work, the authors registered that percentages of waste for meat were 36.4%, for fish were 41.6%, and for eggs were 40.8%, representing a huge loss of important nutrients, like protein, fat, carbohydrates, and fibre. Additionally, these losses represent around 40% of the money spent to produce the food in one day. Many studies were conducted to address the problem of FW in canteens and restaurants (Table 2), confirming that this sector encompasses huge challenges in implementing effective strategies to diminish FW, as well as its economic impact, in addition to social and environmental impacts. Reducing FW at school and university canteens, as well as in hotels and restaurants, is pivotal to ensure proper utilisation of resources, while promoting the preservation of the environment and at the same time contributing to economic gains.

## 7. Food Waste at the Household Level

Developed countries have the highest levels of FW associated with end consumers [13]. Household FW accounts for the largest share of total waste and is associated with environmental problems and monetary losses worldwide [100]. Waste from businesses and households consists mainly of spoiled, uneaten, or inedible food, which in 2020–2021 represented 4.6 Mt [3]. Around 83% of this waste is disposed of in landfills, while a small minority is recycled, resulting in the production of around 8.7 Mt of greenhouse gases [3]. FW during consumption in Australia is around 180–190 kg per capita every year [3]. In South and Southeast Asia, FW per consumer varies between 6 and 11 kg per year, while in Europe and North America, these values are 95 to 115 kg per year [18].

In the case of meat products alone, 22% of meat waste in the United States occurs between sale and delivery, while in the European Union, this figure is 14.5% [19,101]. Regarding poultry waste, consumers in the United States were responsible for 37% of the waste, corresponding to 69.1 billion USD [80]. However, a study carried out in Canada showed that between 1961 and 2009, of the total waste, 40.74% corresponded to poultry waste and 39.73% to red meat [102]. Denmark generates 34,000 tonnes of meat and by-products that are wasted between sale and household consumption [24]. In Iran, 2% to 3% of meat losses are generated at the level of consumption [18]. Australians waste 637.5 million USD worth of meat and fish annually [103]. Still, consumers in the United States are responsible for most of the fish waste, and this trend is increasing [13].

Eggs are a food commodity that generates 50,000 tonnes of shells annually [13]. In Switzerland, 64% of egg waste is produced by consumers, while in the United States, this figure is 14% [27]. Eggshells contain a high amount of calcium carbonate, which can have several applications, including immobilising heavy metals (chromium (III) and lead) in wastewater, contributing to a significant reduction in environmental pollution [104].

Currently, there is also an excessive consumption of food, which also generates waste. This is called metabolic FW [105] and refers to food consumed in excess of physiological needs that results in obesity or overweight, while wasting resources used to produce the food consumed in excess. Europe and North America lead both in excessive consumption and in the resulting waste, largely due to the availability of high-calorie foods, namely meat, eggs, and dairy products [105]. This concept is responsible for generating 140.7 Mt of FW, with Europe being the largest contributor with 39.2 Mt, then North America and Oceania with 32.5 Mt, followed by Latin America with the production of 20 Mt, and finally Sub-Saharan Africa, with around 5 Mt [106]. The reasons for this type of waste are mainly due to a shortage of education, proper information, and knowledge about individual energy needs, which translates into the desire to buy and eat excessively. This, combined with a lack of awareness while eating, such as eating while walking, working or watching television, or a lack of control over food choices and preferences for specific food groups, results in FW. These attitudes towards FW end up generating negative emotional responses such as guilt and sadness when throwing food away [107,108,109,110].

FW in households occurs at all five stages of the consumer decision-making process: planning, purchasing, storage, preparation, and consumption [111,112].

### 7.1. Food Purchasing

**Figure 11:**
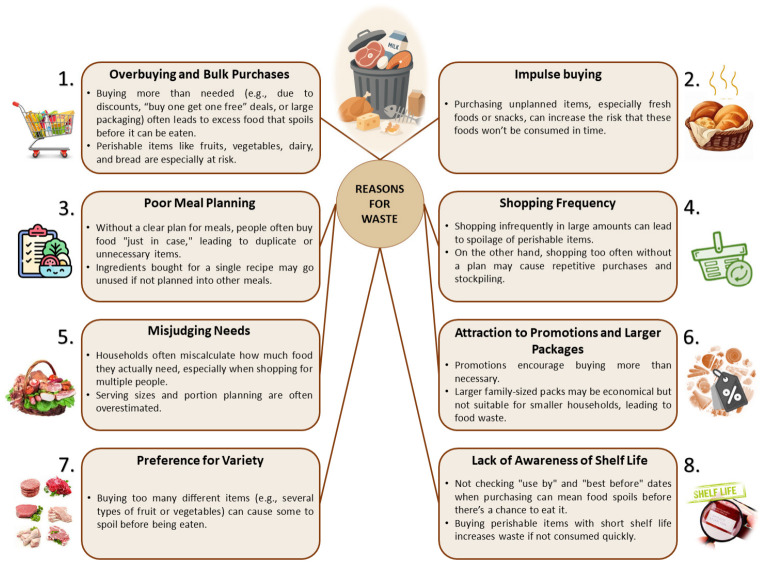
Food purchase influence on food waste at the household level.

Recent evidence confirms that consumer behaviour, particularly purchasing habits, planning practices, and food management routines, is fundamental to understanding and reducing food waste in households [113]. Food purchasing plays a major role in how much food is wasted in households. The way people buy food affects not only what they eat but also what gets thrown away. In fact, household food waste often starts at the point of purchase. Better meal planning, realistic shopping lists, awareness of storage and shelf life, and avoiding overbuying can significantly reduce waste (Figure 11).

A major cause of household FW is poor planning. Even before food arrives at home, there should be planning in order to decide what should or should not be purchased [7]. Planning meals and drawing up a shopping list with the products and quantities in which they should be purchased helps to reduce FW [100,114]. In fact, planning prevents the purchase of excess food, random purchases, and leaving extra food to rot, contributing to the reduction in their respective carbon footprints [100,115]. However, planning may not be effective if the consumer is unable to buy only the products on the list, making unnecessary or impulsive purchases [7,100]. This planning may also not have the desired effect, as families tend to buy the same foods every week, leading to an accumulation of products at home and the subsequent discarding of the oldest ones to accommodate the most recent ones [7,116]. If consumers take stock of the food they still have at home and make a shopping list based on that information, this could be a good way to avoid excessive food purchases [7,100]. In fact, studies show that families with good planning skills tend to waste less food [100]. It was found that families that shopped once a week and assessed the food available at home to make a shopping list and plan meals had a 10% lower level of waste [100].

The act of shopping and the way it is done also influences the generation of FW [7]. Also, the temptation of more attractive prices, such as clearance offers, leads to an increase in waste [117]. Consumers can be easily impressed by promotions or discounts when purchasing large quantities, leading customers to purchase food they do not really need [118]. Lack of management skills also leads to less efficient purchasing and consequent FW [100]. The same is true for families that go shopping more than once a week, as this is associated with over-purchasing and more waste [114,119,120].

Frequent shopping may also be related to the avoidance of uncertainty, which also results in FW [100]. This behaviour is justified by the need to ensure a stock of food at home for any eventuality [100]. A clear example of this was panic buying during the COVID-19 pandemic, where consumers, out of aversion to uncertainty, bought and stored excessive amounts of food [121].

### 7.2. Food Storage at Home

The way food is stored is also a determining factor in household FW. Storage influences how consumers assess the stock and shelf life of food they have at home [116]. Thus, proper food storage leads to lower levels of FW, as it extends the shelf life of food [121,122,123,124]. When these conditions are not guaranteed, waste can be generated, despite all the technology that exists today [105]. In 2020, a study carried out based on the behaviour of German consumers revealed that less than half of consumers ensure the recommended temperatures in refrigerators, leading to 4% of fresh fish and meat and between 15% and 35% of prepared food being wasted [105]. Prolonged storage is another key factor in food being discarded [7]. However, it is not always easy for consumers to understand the appropriate way to preserve food, and there is even contradictory information [7]. In order to clarify the correct methods to adopt, measures such as improved labels, data, and recommendations, as well as technological improvements, should be implemented.

FW is largely influenced by how food is stored and whether it is visible for timely consumption, so small changes can help address this problem [7,116,122]. Packaging, which serves to preserve food and inform the consumer, also plays a major role in food disposal [7].

### 7.3. Preparation of Meals

Household food consumption behaviour obviously influences FW. Behaviours such as saving and eating leftovers or using leftovers to prepare a new meal contribute to reducing FW. The ability to cook creatively is also important, as these people have the ability to create new, equally tasty dishes, using leftovers in a timely manner and combating waste. The use of some small household appliances, such as toasters or blenders, also allows us to reinvent and transform “old” foods into more appreciated ones [116,120].

### 7.4. Consumer Attitudes

Consumers are the main contributors to household FW, but households are poorly aware of the impacts of FW, and around 40% underestimate the amount of food wasted [13,16]. In reality, FW has social implications, in addition to the environmental and economic impacts already described [88]. Although people in general tend to feel unwell about wasting food, the levels of FW are influenced by other factors such as attitude, awareness, behavioural influence, accountability, and social standards [114]. Awareness of the problem also influences the attitude, behaviour, and intentions of each individual [125]. Perceived behavioural control refers to how much individuals believe they can perform a specific behaviour, based on internal determinants, like knowledge and self-efficacy, but also on external factors, like, for example, the availability of waste reduction resources or the existence of regulatory policies, among others [126]. Since consumers do not understand the impact of their behaviours when wasting food, they end up generating waste [7].

At the cultural level, food safety, cultural norms (serving enough food to families), and managing leftovers influence waste [100]. For some consumers, eating foods that may generate distrust or a lack of food safety is not an option, and they end up being discarded [78,127]. In some cultures, there is a habit of preparing a wide variety and quantity of dishes to please guests, which ends up generating more waste [100]. On the contrary, in other cultures, wasting food is a sinful act, given the number of people who die of hunger, and could dictate reducing waste [100].

Sociodemographic factors such as age, family size and composition, and income are also associated with FW [100]. Studies are not consensual, as while some support that larger families waste less [9], others argue the opposite [128], but families with children generate more waste [9].

Regarding family income, and contrary to what would be expected, families with lower incomes tend to waste more food, due to a lack of planning ability [100].

Many studies have described possible ways to avoid FW at home [18]. It is important to emphasise that in order to initiate change on their own initiative, families must be aware that there must be a change in problematic behaviours [124].

Emotional appeal campaigns also tend to be effective, as individuals end up feeling guilty about the act of waste. However, these campaigns are only effective when the individual shows concern about the problem [124]. Currently, the use of influence through social networks has shown positive results [116]. There is a tendency for people to adopt the behaviour of their social group, so if they feel this motivation, they tend to waste less food [116].

Table 3 presents some studies that analysed the role of consumers in the generation of/reduction in FW. Consumers’ behaviour in terms of buying food items, storing them properly at home, or consuming/discarding them is a major factor that needs to be analysed from multiple viewpoints. Education, perceptions, attitudes, and actions of consumers can vary hugely and should be considered when designing and implementing strategies for FW reduction.

Recent studies have found that household food waste also represents a large environmental and economic burden, so substantial reductions are possible through improved food management practices and behavioural changes [115].

## 8. Conclusions, Limitations, and Perspectives

Food waste (FW) associated with animal-derived food products remains a critical challenge at all stages of the food supply chain, from primary production to household consumption. While representing only a fraction of the total volume of food waste in some cases, meat, dairy, egg, and seafood waste have a high environmental, economic, and social impact due to the intensive use of land and water resources, as well as energy and other resources required for their production.

This review provides a comprehensive overview of FW specifically related to animal-derived foods, analysing its impact throughout the supply chain, the main factors contributing to FW at each stage, and the associated environmental, economic, and public health implications. Furthermore, it highlights emerging strategies for mitigating and adding value to by-products, particularly those aligned with the principles of the circular economy. By-product recovery, recycling for animal feed, extraction of bioactive compounds, and industrial reuse of proteins, collagen, and lipids are some emerging solutions. An important contribution of this work is the help in identifying critical points for intervention and more sustainable waste management strategies.

Although this work allowed a systematisation of valuable information regarding the FW of animal origin, it is still relevant to highlight some possible limitations associated with this revision of the literature. We have noticed that this field is characterised by significant methodological heterogeneity and a scarcity of comparable data, which make it difficult to retrieve the appropriate sources and to draw definitive conclusions.

Studies use different definitions for concepts such as food loss, food waste, by-products, co-products, valuable residues, and avoidable/unavoidable waste. This inconsistency makes it difficult to select appropriate keywords for the search of the scientific literature and to conduct robust quantitative syntheses, while also limiting direct comparison of results. Therefore, this absence of universally accepted definitions for food waste and for animal by-product streams limits the comparability of studies and can introduce ambiguities in the interpretation of results.

One other limitation is related to the variability of the methodological approach in the studies analysed (life cycle analyses, economic evaluations, case studies, modelling, surveys, mass balances), making it difficult to compare results and identify consistent global trends.

It is also worth mentioning that in many countries, especially emerging economies, data on losses and waste of animal products are scarce, incomplete, or based on estimates. These sometimes rely mostly on secondary data and may underrepresent some regions in Africa, Latin America, and parts of Asia. Research tends to focus on Europe, North America, and China, while other regions with distinct food systems remain understudied. Allied to this, the literature often emphasises beef, poultry, and dairy products, while other sectors, such as artisanal fishing, aquaculture, sheep, goats, or insects for food, receive less attention.

Future research should focus on identifying edible and non-edible fractions of by-products generated along the food supply chain to improve their utilisation. Secondly, more research is needed to evaluate scalable and economically viable technologies for converting animal-derived food waste into high-value-added products, including functional ingredients, biomaterials, biofuels, and pharmaceutical compounds. Digital tools, such as smart packaging and real-time monitoring systems, should be further explored to improve forecasting, traceability, and waste prevention throughout the supply chain.

It should be emphasised that, throughout the entire process, consumer behaviour and decision-making processes related to food waste must be taken into account, particularly in households and food service establishments, where behavioural interventions can significantly reduce avoidable waste. These measures will surely diminish the pressure on the environment while generating economic profit and positively impacting society.

## Figures and Tables

**Table 1:** Food waste at the processing stages.

Study	Focus	Main Outcomes
Luo et al. [51]	Valorisation of enzymes recovered from animal by-products	Animal by-products constitute a rich source of various functional enzymes that can be recovered and purified to be used in transformation industries, contributing to economic and environmental sustainability.
Dou et al. [52]	Upcycling FW to animal feed	It is important to upcycle many food waste sources (like milk, eggs, fish, or meat residues) to produce animal feed as a strategy to support sustainable production.
Shurson et al. [53]	Regulations regarding the utilisation of FW as animal feed	Laws and regulations vary according to regions, and they either promote or restrict the use of FW in animal feeds. A greater utilisation of safe FW in animal feed involves revising existing regulations.
Jia et al. [54]	Recovery of livestock processing waste to obtain bioplastics	Slaughterhouse waste can be transformed into high-value bioplastics, promoting the circular economy and increasing the sustainability of meat production systems.
Bułkowska et al. [55]	Recovery of products from livestock blood waste	Livestock blood waste can be used to obtain valuable products, including biogas, proteins, haem iron, and bioactive peptides, reducing the negative environmental impact of the blood.
Arena et al. [56]	Valorisation of Mediterranean crabs	The shell, exoskeleton, and cephalothorax of crabs have valuable compounds that can be extracted for industrial use.
Nanda et al. [57]	Valorisation of crab wastes	Crab meat is a rich source of important nutrients, and its processing wastes can be valorised for application in many fields, including the crab shells.

**Table 2:** Food waste in collective catering and restaurants.

Study	Focus	Main Outcomes
Abreu et al. [92]	Analysis of the nutritional and economic impact of FW in a school canteen	Children in the 1st to 9th year of school wasted more food than preschool children, discarding both soup and the main dish. Fish and eggs showed higher percentages of losses than meat.
Li et al. [93]	Reducing FW at university canteens	Results showed that the economic impact and understanding of FW have a major effect on FW among university students. It was further shown that increasing awareness and positive attitudes toward FW is a valuable strategy to reduce FW.
Asefi et al. [94]	Reduction in FW in a Chinese university canteen	The results demonstrated that strategies to reduce FW can bear notable economic benefits by reducing direct food costs while also decreasing expenses associated with waste management and energy costs.
Pizzo et al. [95]	Reducing FW in a restaurant	The results showed that emphasising the problem of waste before ordering food reduces leftovers in restaurants. However, it was observed that providing portion size information did not particularly affect plate waste.
Sigala et al. [96]	Reducing FW in the HORECA sector using AI tools	A total FW of 76–152 g/meal was observed for the HORECA (hotels, restaurants, cafés) under study, representing between 45% and 73% of avoidable FW. The use of the AI tool KITRO allowed FW to be reduced by 23 (51%).
Kostensalo et al. [97]	Proposition of a monitoring program for FW in the Finnish food service sector	The results indicated that food service type is unquestionably the most important factor, explaining both edible and total FW. Other factors of importance were found to be monthly variation, municipal-level variation, and weekday variation, while the outlet size was relatively unimportant.
Basit et al. [98]	Measuring FW from hotel restaurants and caterers in Pakistan	The study evidences that customer plate leftovers are the primary source of FW, being quantified as 58g per customer in restaurants and 150 g in hotels.
Lévesque et al. [99]	Analysis of factors that influence the implementation of FW reduction strategies in restaurants	Several factors are evidenced that affect FW reduction strategies. Also, the adoption of the most suitable FW reduction strategies can lead to higher eco-efficiency in restaurants, although the factors vary according to each restaurant’s characteristics.

**Table 3:** Recent studies on consumers and FW.

Study	Focus	Main Outcomes
Pu et al. [129]	Influence of social media on consumers’ FW	While compulsive buying and obsessive–compulsive buying lead to increased FW, the usage of social media contributes to reduced FW.
Jahan et al. [130]	Factors influencing consumer awareness for reducing FW at home and restaurants	Efforts to reduce FW were found to be the most influential factor, while improper meal storage was demonstrated to be highly associated with FW.
Nguyen et al. [131]	Sustainability versus nutrition motivators for FW reduction	Consumers with nutrition consciousness tend to avoid FW by better planning and more adequate shopping behaviours, unlike consumers with sustainability consciousness, who do not significantly diminish FW.
Cliceri et al. [132]	Consumer attitudes according to date labels and implications for FW	Understanding of ‘best before’ and ‘use by’ mentions was high among European consumers. Utilisation of goods after the expiration date was found to be related to a reduction in household FW, and some consumers eat recently expired foods to reduce FW.
Imran et al. [133]	Consumer behaviour towards carbon-labelled food products	Results indicated a gap between consumer awareness and behavioural change. While two-thirds of consumers recognise the importance of carbon labelling, only one-third shape their buying decisions in accordance with carbon labelling.
Bytyqi et al. [134]	Consumer attitudes concerning animal-derived FW	Strategies identified as leading to a reduction in FW include education, clearer labelling, and policy changes.
Tadesse et al. [135]	Consumer choices related to innovative food preservation techniques	Consumers tend to value active packaging and temperature indicators that monitor food freshness in fish and meat products. Also, consumers prize traceability and natural preservation techniques.
Cruecha-Em et al. [136]	Circular economy of food packaging	Consumers value recyclable and biodegradable food packaging materials and value a reuse model, being willing to pay for these solutions.

## Data Availability

No new data were created or analysed in this study. Data sharing is not applicable to this article.

## References

[1] FAO Introduction—Food Loss|Technical Platform on the Measurement and Reduction of Food Loss and Waste. https://www.fao.org/platform-food-loss-waste/food-loss/introduction/en.

[2] Olabode O., Kumar N., De D. (2025). Food Loss and Waste Management in the Retail Food Supply Chain: Methods and Framework to Achieve Environmental Sustainability. J. Environ. Manag..

[3] Talekar S., Ekanayake K., Holland B., Barrow C. (2023). Food Waste Biorefinery towards Circular Economy in Australia. Bioresour. Technol..

[4] Teigiserova D.A., Hamelin L., Thomsen M. (2020). Towards Transparent Valorization of Food Surplus, Waste and Loss: Clarifying Definitions, Food Waste Hierarchy, and Role in the Circular Economy. Sci. Total Environ..

[5] Uhlig E., Sadzik A., Strenger M., Schneider A.-M., Schmid M. (2025). Food Wastage along the Global Food Supply Chain and the Impact of Food Packaging. J. Consum. Prot. Food Saf..

[6] Patel A., Hrůzová K., Rova U., Christakopoulos P., Matsakas L. (2019). Sustainable Biorefinery Concept for Biofuel Production through Holistic Volarization of Food Waste. Bioresour. Technol..

[7] Haldar D., Shabbirahmed A.M., Singhania R.R., Chen C.-W., Dong C.-D., Ponnusamy V.K., Patel A.K. (2022). Understanding the Management of Household Food Waste and Its Engineering for Sustainable Valorization- A State-of-the-Art Review. Bioresour. Technol..

[8] Antasouras G., Vasios G.K., Kontogiorgis C., Ioannou Z., Poulios E., Deligiannidou G.-E., Troumbis A.Y., Giaginis C. (2023). How to Improve Food Waste Management in Hospitals through Focussing on the Four Most Common Measures for Reducing Plate Waste. Int. J. Health Plan. Manag..

[9] Parfitt J., Barthel M., Macnaughton S. (2010). Food Waste within Food Supply Chains: Quantification and Potential for Change to 2050. Philos. Trans. R. Soc. B Biol. Sci..

[10] Lipinski B., Hanson C., Lomax J., Kitinoja L., Waite R., Searchinger T. (2013). Reducing Food Loss and Waste. World Resour. Inst..

[11] Gustavson J., Cederberg C., Sonesson U., Otterdijk R., Meybeck A. (2011). Global Food Losses and Food Waste—Extent, Causes and Preventio.

[12] FAO Food Loss and Waste Facts 2015. https://openknowledge.fao.org/server/api/core/bitstreams/82e4fdce-6cbb-4837-a615-e249e876acc1/content.

[13] Ghosh P.R., Fawcett D., Sharma S.B., Poinern G.E.J. (2016). Progress towards Sustainable Utilisation and Management of Food Wastes in the Global Economy. Int. J. Food Sci..

[14] Buzby J.C., Hyman J. (2012). Total and per Capita Value of Food Loss in the United States. Food Policy.

[15] Redlingshöfer B., Soyeux A. Food Losses and Wastage as a Sustainability Indicator of Food and Farming Systems. Proceedings of the 10th European IFSA Symposium.

[16] Mak T.M.W., Xiong X., Tsang D.C.W., Yu I.K.M., Poon C.S. (2020). Sustainable Food Waste Management towards Circular Bioeconomy: Policy Review, Limitations and Opportunities. Bioresour. Technol..

[17] Parmenter B.H., Thompson A.S., Bondonno N.P., Jennings A., Murray K., Perez-Cornago A., Hodgson J.M., Tresserra-Rimbau A., Kühn T., Cassidy A. (2025). High Diversity of Dietary Flavonoid Intake Is Associated with a Lower Risk of All-Cause Mortality and Major Chronic Diseases. Nat. Food.

[18] Ranaei V., Pilevar Z., Esfandiari C., Khaneghah A.M., Dhakal R., Vargas-Bello-Pérez E., Hosseini H. (2021). Meat Value Chain Losses in Iran. Food Sci. Anim. Resour..

[19] Den Toorn S.I.A., Tziva M., van den Broek M., Negro S.O., Hekkert M.P., Worrell E. (2018). Climate Innovations in Meat and Dairy.

[20] Kayikci Y., Ozbiltekin M., Kazancoglu Y. (2020). Minimizing Losses at Red Meat Supply Chain with Circular and Central Slaughterhouse Model. J. Enterp. Inf. Manag..

[21] Jayathilakan K., Sultana K., Radhakrishna K., Bawa A.S. (2012). Utilization of Byproducts and Waste Materials from Meat, Poultry and Fish Processing Industries: A Review. J. Food Sci. Technol..

[22] Berton M., Sturaro E., Schiavon S., Bittante G., Cecchinato A., Xiccato G., Gallo L. (2025). Biogenic and Fossil Main Greenhouse Gas Emissions of Dairy, Beef, Pig and Poultry Systems. Animal.

[23] Guiné R.P.F., Correia P., Coelho C., Costa C.A. (2021). The Role of Edible Insects to Mitigate Challenges for Sustainability. Open Agric..

[24] Halloran A., Clement J., Kornum N., Bucatariu C., Magid J. (2014). Addressing Food Waste Reduction in Denmark. Food Policy.

[25] Ghosh R. (2001). Fractionation of Biological Macromolecules Using Carrier Phase Ultrafiltration. Biotechnol. Bioeng..

[26] Cuéllar A.D., Webber M.E. (2010). Wasted Food, Wasted Energy: The Embedded Energy in Food Waste in the United States. Environ. Sci. Technol..

[27] Beretta C., Stoessel F., Baier U., Hellweg S. (2013). Quantifying Food Losses and the Potential for Reduction in Switzerland. Waste Manag..

[28] Theron H., Venter P., Lues J.F.R. (2003). Bacterial Growth on Chicken Eggs in Various Storage Environments. Food Res. Int..

[29] Arunlertaree C., Kaewsomboon W., Kumsopa A., Pokethitiyook P., Panyawathanakit P. (2007). Removal of Lead from Battery Manufacturing Wastewater by Egg Shell. Songklanakarin J. Sci. Technol..

[30] Park H.J., Jeong S.W., Yang J.K., Kim B.G., Lee S.M. (2007). Removal of Heavy Metals Using Waste Eggshell. J. Environ. Sci..

[31] Hospido A., Moreira M.T., Feijoo G. (2003). Simplified Life Cycle Assessment of Galician Milk Production. Int. Dairy J..

[32] Roy P., Nei D., Orikasa T., Xu Q., Okadome H., Nakamura N., Shiina T. (2009). A Review of Life Cycle Assessment (LCA) on Some Food Products. J. Food Eng..

[33] FAO (2012). The State of World Fisheries and Aquaculture.

[34] Rolf W., Kieran K. (2009). The Sunken Billions: The Economic Justification for Fisheries Reform.

[35] (2013). World Bank Fish to 2030: Prospects for Fisheries and Aquaculture.

[36] Archer M. (2001). Fish Waste Production in the United Kingdom.

[37] Costello C., Ovando D., Hilborn R., Gaines S.D., Deschenes O., Lester S.E. (2012). Status and Solutions for the World’s Unassessed Fisheries. Science.

[38] Pugliese G., Puvača N., Passantino L., Perillo A., Laudadio V., Tateo A., Piemontese L., Dimuccio M.M., Lauriola S., Tufarelli V. (2026). Drawing a Circle for the Livestock and Agrifood Sector: Fundamentals to a Sustainable Supply Chain. Front. Anim. Sci..

[39] Panesar R., Kaur S., Panesar P.S. (2015). Production of Microbial Pigments Utilizing Agro-Industrial Waste: A Review. Curr. Opin. Food Sci..

[40] Liu J., Lundqvist J., Weinberg J., Gustafsson J. (2013). Food Losses and Waste in China and Their Implication for Water and Land. Environ. Sci. Technol..

[41] Sharma P., Gaur V.K., Gupta S., Varjani S., Pandey A., Gnansounou E., You S., Ngo H.H., Wong J.W.C. (2022). Trends in Mitigation of Industrial Waste: Global Health Hazards, Environmental Implications and Waste Derived Economy for Environmental Sustainability. Sci. Total Environ..

[42] Osorio L.L.D.R., Flórez-López E., Grande-Tovar C.D. (2021). The Potential of Selected Agri-Food Loss and Waste to Contribute to a Circular Economy: Applications in the Food, Cosmetic and Pharmaceutical Industries. Molecules.

[43] Vendruscolo F., Albuquerque P.M., Streit F., Esposito E., Ninow J.L. (2008). Apple Pomace: A Versatile Substrate for Biotechnological Applications. Crit. Rev. Biotechnol..

[44] Nikmaram N., Rosentrater K.A. (2019). Overview of Some Recent Advances in Improving Water and Energy Efficiencies in Food Processing Factories. Front. Nutr..

[45] Kumla J., Suwannarach N., Sujarit K., Penkhrue W., Kakumyan P., Jatuwong K., Vadthanarat S., Lumyong S. (2020). Cultivation of Mushrooms and Their Lignocellulolytic Enzyme Production Through the Utilization of Agro-Industrial Waste. Molecules.

[46] Gaur V.K., Sharma P., Sirohi R., Awasthi M.K., Dussap C.-G., Pandey A. (2020). Assessing the Impact of Industrial Waste on Environment and Mitigation Strategies: A Comprehensive Review. J. Hazard. Mater..

[47] Caldeira C., Vlysidis A., Fiore G., De Laurentiis V., Vignali G., Sala S. (2020). Sustainability of Food Waste Biorefinery: A Review on Valorisation Pathways, Techno-Economic Constraints, and Environmental Assessment. Bioresour. Technol..

[48] Correddu F., Lunesu M.F., Buffa G., Atzori A.S., Nudda A., Battacone G., Pulina G. (2020). Can Agro-Industrial By-Products Rich in Polyphenols Be Advantageously Used in the Feeding and Nutrition of Dairy Small Ruminants?. Animals.

[49] Coppola D., Lauritano C., Palma Esposito F., Riccio G., Rizzo C., de Pascale D. (2021). Fish Waste: From Problem to Valuable Resource. Mar. Drugs.

[50] Das M., Ghosh S. (2016). Comparative Study of Whey Utilization in India, New Zealand and Australia-Identifying Untapped Potential and Means of Utilization. J. Solid Waste Technol. Manag..

[51] Luo W., Zhang J., Ahmmed M.K., Sakai K., Shahidi F., Zhi Z., Wu H. (2025). Valorization of Animal By-Product Enzymes: Advancing Sustainable Food Processing through Innovative Extraction, Purification, and Application Strategies. Trends Food Sci. Technol..

[52] Dou Z., Dierenfeld E.S., Wang X., Chen X., Shurson G.C. (2024). A Critical Analysis of Challenges and Opportunities for Upcycling Food Waste to Animal Feed to Reduce Climate and Resource Burdens. Resour. Conserv. Recycl..

[53] Shurson G.C., Dierenfeld E.S., Dou Z. (2023). Rules Are Meant to Be Broken—Rethinking the Regulations on the Use of Food Waste as Animal Feed. Resour. Conserv. Recycl..

[54] Jia C., Wang S., Wang L. (2025). Upcycling Livestock Processing Waste to Bioplastics for Sustainable Meat Production Systems: Material Quantification and Environmental Impact. Resour. Conserv. Recycl..

[55] Bułkowska K., Zielińska M. (2024). Recovery of Biogas and Other Valuable Bioproducts from Livestock Blood Waste: A Review. Energies.

[56] Arena R., Renda G., Ottaviani Aalmo G., Debeaufort F., Messina C.M., Santulli A. (2024). Valorization of the Invasive Blue Crabs (*Callinectes sapidus*) in the Mediterranean: Nutritional Value, Bioactive Compounds and Sustainable By-Products Utilization. Mar. Drugs.

[57] Nanda P.K., Das A.K., Dandapat P., Dhar P., Bandyopadhyay S., Dib A.L., Lorenzo J.M., Gagaoua M. (2021). Nutritional Aspects, Flavour Profile and Health Benefits of Crab Meat Based Novel Food Products and Valorisation of Processing Waste to Wealth: A Review. Trends Food Sci. Technol..

[58] Sivalingam S., Nandhitha S., Pragadheeshwaran S.T. (2026). A Comprehensive Review on Cradle to Cradle Strategies for Sustainable Food Waste Valorization. Discov. Food.

[59] Goldáraz-Salamero N., Blanc S., Sierra-Perez J., Brun F. (2025). From Food Loss and Waste to Feed: A PRISMA of Life Cycle Perspectives in Livestock Systems. Int. J. Life Cycle Assess.

[60] Dey S., Talukdar A., Bhattacharya S. (2025). Microbial Degradation and Valorization of Food Wastes: Waste to Wealth Approaches towards Sustainability. Discov. Chem..

[61] Hosseini H., Cheraghali A.M., Yalfani R., Razavilar V. (2004). Incidence of *Vibrio* Spp. in Shrimp Caught off the South Coast of Iran. Food Control.

[62] Akramzadeh N., Ramezani Z., Ferdousi R., Akbari-Adergani B., Mohammadi A., Karimian-khosroshahi N., Famenin B.K., Pilevar Z., Hosseini H. (2020). Effect of Chicken Raw Materials on Physicochemical and Microbiological Properties of mechanically Deboned Chicken Meat. Vet. Res. Forum.

[63] Ünsal M., Aktaş N. (2003). Fractionation and Characterization of Edible Sheep Tail Fat. Meat Sci..

[64] Kyakuwaire M., Olupot G., Amoding A., Nkedi-Kizza P., Ateenyi Basamba T. (2019). How Safe Is Chicken Litter for Land Application as an Organic Fertilizer?: A Review. Int. J. Environ. Res. Public Health.

[65] Singh N.B., Singh R., Imam M.M. (2014). Waste Water Management in Dairy Industry: Pollution Abatement and Preventive Attitudes. Int. J. Sci. Environ. Technol..

[66] Knuckey I., Sinclair C., Surapaneni A., Ashcroft W. Utilisation of Seafood Processing Waste—Challenges and Opportunities. Proceedings of the 3rd Australian New Zealand Soils Conference.

[67] Gumisiriza R., Mshandete A., Steven M., Rubindamayugi M., Kansiime F., Kivaisi A. (2009). Nile Perch Fish Processing Waste along Lake Victoria in East Africa: Auditing and Characterization. Afr. J. Environ. Sci. Technol..

[68] Yan N., Chen X. (2015). Sustainability: Don’t Waste Seafood Waste. Nature.

[69] Gupta S., Pawar S.B. (2018). An Integrated Approach for Microalgae Cultivation Using Raw and Anaerobic Digested Wastewaters from Food Processing Industry. Bioresour. Technol..

[70] Armstrong K., Dong W., Jin M., Nimbalkar S., Cresko J. (2025). Estimating Energy Consumption and GHG Emissions in the U.S. Food Supply Chain for Net-Zero. npj Sci. Food.

[71] Love D.C., Fry J.P., Milli M.C., Neff R.A. (2015). Wasted Seafood in the United States: Quantifying Loss from Production to Consumption and Moving toward Solutions. Glob. Environ. Change.

[72] Ramírez-García R., Gohil N., Singh V., Pandey V.C., Bauddh K. (2019). Chapter 21—Recent Advances, Challenges, and Opportunities in Bioremediation of Hazardous Materials. Phytomanagement of Polluted Sites.

[73] Hewitt L.M., Kovacs T.G., Dubé M.G., MacLatchy D.L., Martel P.H., McMaster M.E., Paice M.G., Parrott J.L., van den Heuvel M.R., van der Kraak G.J. (2008). Altered Reproduction in Fish Exposed to Pulp and Paper Mill Effluents: Roles of Individual Compounds and Mill Operating Conditions. Environ. Toxicol. Chem..

[74] Larsson D.G.J., Förlin L. (2002). Male-Biased Sex Ratios of Fish Embryos near a Pulp Mill: Temporary Recovery after a Short-Term Shutdown. Environ. Health Perspect..

[75] Kosseva M.R., Moo-Young M. (2011). 6.44—Management and Processing of Food Wastes. Comprehensive Biotechnology (Second Edition).

[76] Gustavsson J., Stage J. (2011). Retail Waste of Horticultural Products in Sweden. Resour. Conserv. Recycl..

[77] Pinto J., Boavida-Dias R., Matos H.A., Azevedo J. (2022). Analysis of the Food Loss and Waste Valorisation of Animal By-Products from the Retail Sector. Sustainability.

[78] Graham-Rowe E., Jessop D.C., Sparks P. (2014). Identifying Motivations and Barriers to Minimising Household Food Waste. Resour. Conserv. Recycl..

[79] Wilson N.L.W., Rickard B.J., Saputo R., Ho S.-T. (2017). Food Waste: The Role of Date Labels, Package Size, and Product Category. Food Qual. Prefer..

[80] Mena C., Adenso-Diaz B., Yurt O. (2011). The Causes of Food Waste in the Supplier–Retailer Interface: Evidences from the UK and Spain. Resour. Conserv. Recycl..

[81] Ehtesabi H., Afkaneh S.Z. (2025). Smartphone-Based Label on Package for Monitoring the Freshness of Meat: A Review. Food Res. Int..

[82] Thies A.J., Holloway M., Altmann B.A., Countryman A.M., Smith C., Nair M.N. (2025). Addressing Retail Meat Waste: A Focus Group Study on Discolored Beef. Food Humanit..

[83] Lebersorger S., Schneider F. (2014). Food Loss Rates at the Food Retail, Influencing Factors and Reasons as a Basis for Waste Prevention Measures. Waste Manag..

[84] Thyberg K.L., Tonjes D.J. (2016). Drivers of Food Waste and Their Implications for Sustainable Policy Development. Resour. Conserv. Recycl..

[85] Betz A., Buchli J., Göbel C., Müller C. (2015). Food Waste in the Swiss Food Service Industry—Magnitude and Potential for Reduction. Waste Manag..

[86] Engström R., Carlsson-Kanyama A. (2004). Food Losses in Food Service Institutions Examples from Sweden. Food Policy.

[87] Busetti S. (2019). A Theory-Based Evaluation of Food Waste Policy: Evidence from Italy. Food Policy.

[88] Papargyropoulou E., Lozano R., Steinberger J.K., Wright N., bin Ujang Z. (2014). The Food Waste Hierarchy as a Framework for the Management of Food Surplus and Food Waste. J. Clean. Prod..

[89] Schneider F. (2013). The Evolution of Food Donation with Respect to Waste Prevention. Waste Manag..

[90] Oroski F.d.A. (2025). Understanding Food Surplus: Challenges and Strategies for Reducing Food Waste—A Mini-Review. Waste Manag. Res..

[91] Yang Y., Wang X., Qi S., Zhuang M. (2025). Spatial Disparity of Life-Cycle Water Use and Greenhouse Gas Emissions from Food Waste Resource Conversion in China. Environ. Impact Assess. Rev..

[92] Abreu M., Carvalho J., Gonçalves C. (2025). Evaluation of Nutritional and Economic Impact of Food Waste in a School Canteen. Meas. Food.

[93] Li Y., Liang Y., Yu D., Xu L., Song Q. (2025). Reducing Food Waste Behaviors from the Viewpoint of University Students through the E-TPB Model. Circ. Econ..

[94] Asefi B., Nkinahamira F., Abass O.K., Maenrouf M.S., Liang Y., Meng Y., Wang P. (2025). Sustainability Analysis and Impact Assessment of Food Waste Reduction Campaign in Chinese Higher Institution: Towards Cleaner Environment and Energy Efficiency. Clean. Eng. Technol..

[95] Pizzo A., Suter M., Bauer J.M., Reisch L.A. (2025). Food Waste Salience and Task Knowledge to Reduce Individual Food Waste: A Field Experiment in a Restaurant Setting. J. Behav. Exp. Econ..

[96] Sigala E.G., Gerwin P., Chroni C., Abeliotis K., Strotmann C., Lasaridi K. (2025). Reducing Food Waste in the HORECA Sector Using AI-Based Waste-Tracking Devices. Waste Manag..

[97] Kostensalo J., Silvennoinen K., Kettunen M., Lampi V. (2025). Optimal Design of National Food Waste Monitoring for the Food Service Sector. J. Clean. Prod..

[98] Basit A., Urooj N., Humza M., Hameed M.S. (2025). A Survey of Quantifying Food Waste from Hotel Restaurants and Caterers in Multan, Southern Punjab, Pakistan. Food Humanit..

[99] Lévesque J., Godin L., Perreault V., Mikhaylin S. (2024). Identifying the Factors Affecting the Implementation of Food Waste Reduction Strategies in Independent Restaurants: Moving towards Eco-Efficiency. J. Clean. Prod..

[100] Ananda J., Karunasena G.G., Mitsis A., Kansal M., Pearson D. (2021). Analysing Behavioural and Socio-Demographic Factors and Practices Influencing Australian Household Food Waste. J. Clean. Prod..

[101] Buzby J.C., Wells H.F., Hyman J. (2014). The estimated amount, value, and calories of postharvest food losses at the retail and consumer levels in the United States. Econ. Inf. Bull..

[102] Abdulla M., Martin R., Gooch M., Jovel E. (2013). The Importance of Quantifying Food Waste in Canada. J. Agric. Food Syst. Community Dev..

[103] Baker D., Fear J., Denniss R. (2009). What a Waste—An Analysis of Household Expenditure on Food.

[104] Ok Y.S., Lee S.S., Jeon W.-T., Oh S.-E., Usman A.R.A., Moon D.H. (2011). Application of Eggshell Waste for the Immobilization of Cadmium and Lead in a Contaminated Soil. Environ. Geochem Health.

[105] Balan I.M., Gherman E.D., Brad I., Gherman R., Horablaga A., Trasca T.I. (2022). Metabolic Food Waste as Food Insecurity Factor—Causes and Preventions. Foods.

[106] Toti E., Di Mattia C., Serafini M. (2019). Metabolic Food Waste and Ecological Impact of Obesity in FAO World’s Region. Front. Nutr..

[107] Serafini M., Toti E. (2016). Unsustainability of Obesity: Metabolic Food Waste. Front. Nutr..

[108] Wucher H., Klingshirn A., Brugger L., Stamminger R., Geppert J., Kölzer B., Engstler A., Härlen J. (2020). Tackling Food Waste: Impact of German Consumer Behaviour on Food in Chilled Storage. Foods.

[109] Przezbórska-Skobiej L., Wiza P.L. (2021). Food Waste in Households in Poland—Attitudes of Young and Older Consumers towards the Phenomenon of Food Waste as Demonstrated by Students and Lecturers of PULS. Sustainability.

[110] Althumiri N.A., Basyouni M.H., Duhaim A.F., AlMousa N., AlJuwaysim M.F., BinDhim N.F. (2021). Understanding Food Waste, Food Insecurity, and the Gap between the Two: A Nationwide Cross-Sectional Study in Saudi Arabia. Foods.

[111] Schanes K., Dobernig K., Gözet B. (2018). Food Waste Matters—A Systematic Review of Household Food Waste Practices and Their Policy Implications. J. Clean. Prod..

[112] Pearson D., Mirosa M., Andrews L., Kerr G. (2017). Reframing Communications That Encourage Individuals to Reduce Food Waste. Commun. Res. Pract..

[113] Salume P.K., Barbosa M.W., Pinto M.d.R. (2026). Food Waste and Consumer Behavior: A Bibliometric and Review Study and Future Research Directions. Foods.

[114] Stancu V., Haugaard P., Lähteenmäki L. (2016). Determinants of Consumer Food Waste Behaviour: Two Routes to Food Waste. Appetite.

[115] Sjölund A., Sundin N., Svensson E., Aroko Y.P., Eriksson M., Malefors C. (2026). Quantifying the Realistic Reduction Potential of Food Waste in Swedish Households. Sci. Rep..

[116] Geffen L., Herpen E., Trijp H., Närvänen E., Mesiranta N., Mattila M., Heikkinen A. (2020). Household Food Waste—How to Avoid It? An Integrative Review. Food Waste Management: Solving the Wicked Problem.

[117] Roodhuyzen D.M.A., Luning P.A., Fogliano V., Steenbekkers L.P.A. (2017). Putting Together the Puzzle of Consumer Food Waste: Towards an Integral Perspective. Trends Food Sci. Technol..

[118] Pearson D., Perera A. (2018). Reducing Food Waste: A Practitioner Guide Identifying Requirements for an Integrated Social Marketing Communication Campaign. Soc. Mark. Q..

[119] Tucker C.A., Farrelly T. (2016). Household Food Waste: The Implications of Consumer Choice in Food from Purchase to Disposal. Local Environ..

[120] Aschemann-Witzel J., De Hooge I., Amani P., Bech-Larsen T., Oostindjer M. (2015). Consumer-Related Food Waste: Causes and Potential for Action. Sustainability.

[121] Evans D. (2011). Blaming the Consumer—Once Again: The Social and Material Contexts of Everyday Food Waste Practices in Some English Households. Crit. Public Health.

[122] Farr-Wharton G., Foth M., Choi J.H.-J. (2014). Identifying Factors That Promote Consumer Behaviours Causing Expired Domestic Food Waste. J. Consum. Behav..

[123] Quested T.E., Marsh E., Stunell D., Parry A.D. (2013). Spaghetti Soup: The Complex World of Food Waste Behaviours. Resour. Conserv. Recycl..

[124] Geffen L., Herpen E., Sijtsema S., van Trijp H. (2020). Food Waste as the Consequence of Competing Motivations, Lack of Opportunities, and Insufficient Abilities. Resour. Conserv. Recycl. X.

[125] Nilsson D., Fielding K., Dean A.J. (2020). Achieving Conservation Impact by Shifting Focus from Human Attitudes to Behaviors. Conserv. Biol..

[126] Kalogiannidis S., Kalfas D., Papathanasiou F., Chatzitheodoridis F. (2025). Analysis of Food Waste Generation and Its Implications for Food Security and Ecosystem Sustainability in Europe. Sustain. Futures.

[127] Canali M., Amani P., Aramyan L., Gheoldus M., Moates G., Östergren K., Silvennoinen K., Waldron K., Vittuari M. (2017). Food Waste Drivers in Europe, from Identification to Possible Interventions. Sustainability.

[128] Fami H.S., Aramyan L.H., Sijtsema S.J., Alambaigi A. (2019). Determinants of Household Food Waste Behavior in Tehran City: A Structural Model. Resour. Conserv. Recycl..

[129] Pu X., Ma K., Han G. (2025). Reducing Food Waste in Media Age: How Does Social Media Usage Affect Consumers’ Food Waste. Waste Manag..

[130] Jahan I., Kamal K.T., Bhattacharjee P., Taqi H.M.M., Ali S.M. (2025). Improving Consumer Awareness for Reducing Food Waste Using Partial Least Squares Structural Equation Modelling (PLS-SEM) Approach. Clean. Responsible Consum..

[131] Nguyen T.T.T., Hetherington J.B., O’Connor P.J., Malek L. (2025). Sustainable Food Consumption: Sustainability-Conscious Consumers Do Not Reduce Food Waste but Nutrition-Conscious Consumers Do. Resour. Conserv. Recycl..

[132] Cliceri D., Pedrotti M., Gasperi F., Endrizzi I. (2025). European Consumers’ Involvement with Date Labels and Implications for Household Food Waste. Food Qual. Prefer..

[133] Imran N., Kumar M., Jagtap S., Trollman H., Gupta S., Garcia-Garcia G. (2025). Exploring Consumer Behaviour on Carbon Labelled Food Products: Evidence from a Survey on the Case of Sandwich Production and Consumption in UK. J. Agric. Food Res..

[134] Bytyqi H., Ender Kunili I., Mestani M., Adam Antoniak M., Berisha K., Ozge Dinc S., Guzik P., Szymkowiak A., Kulawik P. (2025). Consumer Attitudes towards Animal-Derived Food Waste and Ways to Mitigate Food Loss at the Consumer Level. Trends Food Sci. Technol..

[135] Tadesse T., Ramírez-Rodríguez S., Rahmani D., Gezahegn T.W., Verherbrugghen H., Vermeulen A., Skourletis N., Gil Roig J.M. (2025). Healthy but Also Traceable: Consumer Choices for Traits of Food Waste-Reducing Innovations. J. Clean. Prod..

[136] Cruecha-Em M., Hossain M.J., Nuansri A., Zulfiqar F. (2025). Circular Economy of Food Packaging: Cost and Functional Preferences of Thai Consumers and Restaurants in off-Premises Food Services. Sustain. Futures.

